# The High Cost of Free Tuberculosis Services: Patient and Household Costs Associated with Tuberculosis Care in Ebonyi State, Nigeria

**DOI:** 10.1371/journal.pone.0073134

**Published:** 2013-08-27

**Authors:** Kingsley N. Ukwaja, Isaac Alobu, Chika lgwenyi, Philip C. Hopewell

**Affiliations:** 1 Department of Internal Medicine, Federal Teaching Hospital, Abakaliki, Ebonyi State, Nigeria; 2 National Tuberculosis and Leprosy Control Programme, Ministry of Health, Ebonyi State, Nigeria; 3 Francis J. Curry International Tuberculosis Center, Division of Pulmonary and Critical Care Medicine, San Francisco General Hospital, University of California, San Francisco, California, United States of America; University of Otago, New Zealand

## Abstract

**Objective:**

Poverty is both a cause and consequence of tuberculosis. The objective of this study is to quantify patient/household costs for an episode of tuberculosis (TB), its relationships with household impoverishment, and the strategies used to cope with the costs by TB patients in a resource-limited high TB/HIV setting.

**Methods:**

A cross-sectional study was conducted in three rural hospitals in southeast Nigeria. Consecutive adults with newly diagnosed pulmonary TB were interviewed to determine the costs each incurred in their care-seeking pathway using a standardised questionnaire. We defined direct costs as out-of-pocket payments, and indirect costs as lost income.

**Results:**

Of 452 patients enrolled, majority were male 55% (249), and rural residents 79% (356), with a mean age of 34 (±11.6) years. Median direct pre-diagnosis/diagnosis cost was $49 per patient. Median direct treatment cost was $36 per patient. Indirect pre-diagnostic and treatment costs were $416, or 79% of total patient costs, $528. The median total cost of TB care per household was $592; corresponding to 37% of median annual household income pre-TB. Most patients reported having to borrow money 212(47%), sell assets 42(9%), or both 144(32%) to cope with the cost of care. Following an episode of TB, household income reduced increasing the proportion of households classified as poor from 54% to 79%. Before TB illness, independent predictors of household poverty were; rural residence (adjusted odds ratio [aOR] 2.8), HIV-positive status (aOR 4.8), and care-seeking at a private facility (aOR 5.1). After TB care, independent determinants of household poverty were; younger age (≤35 years; aOR 2.4), male gender (aOR 2.1), and HIV-positive status (aOR 2.5).

**Conclusion:**

Patient and household costs for TB care are potentially catastrophic even where services are provided free-of-charge. There is an urgent need to implement strategies for TB care that are affordable for the poor.

## Introduction

In 2010, more than a quarter of tuberculosis (TB) cases and 23% of TB-related mortality occurred among Africans [1]. Nigeria has one of the highest TB incidence rates in Africa (199 per 100 000 population) [1], and one of the highest TB/human immunodeficiency virus (HIV) co-infection rates (25%) [1]. In Nigeria, the public health-care system, into which TB control is fully integrated, is constrained by lack of human resources and difficulties in providing outreach services particularly in rural areas. And due to the inconsistencies in the quality of care given by for-profit private hospitals throughout the country, they have not been adequately integrated in to the programme [1]. Thus, despite achieving 99% Directly Observed Treatment Short course (DOTS) coverage in 2007 [2], Nigeria ranks 4^th^ among high tuberculosis burden countries in the world with a low case detection rate for all tuberculosis cases of 40% [1].

Universal access to care and reducing the socioeconomic burden associated with tuberculosis are key objectives of the current World Health Organisation (WHO) Stop-TB strategy [3]. The WHO lists economic factors as one of four barriers to tuberculosis care [3]. Economic factors may include medical costs; transport, accommodation and subsistence costs, lost income, productivity and time. Moreover, TB mainly affects the most economically-productive age group, this results in substantial economic burden on affected households [4]. Such economic barriers may limit the use of health services, particularly by the rural poor [5].

Although few studies have assessed the costs of TB care for patients and households in Africa, methodological bottlenecks identified in these studies precluded systematic analysis [6]–[11]. A systematic review of the economic burden of TB care in the region showed that full costs have been assessed only for an urban population in Zambia [12], [13]. Studies of both urban and rural populations reported only average direct or indirect costs covering either the pre-diagnostic or post-diagnostic period only [12]. And, few investigated how patients cope with the costs of tuberculosis care and the potential gains of collaborative TB/HIV services [12]. However, more recently, full patient costs for TB services have been reported for Kenya and Ghana, but it was during a pilot-test for a standardized instrument on patients' costs [14], [15].

In order to establish an evidence base for interventions that can contribute to TB-related poverty reduction, increase equity in accessing care, and increase TB case detection [12], there is need to document the full economic burden of TB care borne by patients across various settings in Africa with a standardised instrument. Nigeria's National TB & Leprosy Control Programme (NTBLCP) has removed user fees, fees for anti-TB drugs and laboratory tests, and introduced a community-based DOTS strategy which has reduced patients' costs [2]. However, costs to patients and their households of care-seeking from illness onset to treatment are less well-documented [16]. We estimated the comprehensive costs of TB diagnosis and treatment (intensive phase) from the patients' perspective in an under-resourced high TB/HIV setting. Specifically, we determined the components of these costs, their relationship to household impoverishment, and the strategies used to cope with the costs.

## Materials and Methods

### Study area

Ebonyi State is located in the South Eastern region of Nigeria with an estimated population of over 2.5 million people [17]. The state is divided into three geopolitical zones each having four or five of the 13 local government areas (LGA) in the state. Although recent data show a decreasing incidence of TB in Ebonyi state [18], case detection rates remain below 40% [unpublished data]. Also, HIV prevalence assessed through national sentinel surveys among pregnant women showed that HIV prevalence in the state has declined from 9.3% (1999) to 3.3% (2010) [19], but TB/HIV co-infection rate is 28% [20].

### Study Setting

Due to constraints of resources and the inability of health system to recruit and retain rural health workers in the public sector, there is limited availability of health services in public facilities in rural and remote areas of Ebonyi State. Thus, several not-for-profit mission (private) hospitals provide basic and secondary health services in those settings. The NTBLCP, with the support of the German Leprosy Relief Association incorporated these rural private facilities for TB service delivery in the state. The NTBLCP provides anti-TB drugs and laboratory services as well as paying the fees for radiologic imaging studies (X-ray) in these facilities. The quality of TB services provided at health facilities covered by the NTBLCP at facility level are monitored by the district and zonal Tuberculosis and Leprosy Control supervisors responsible for managing and coordinating TB and leprosy control activities in their respective districts and zones. Also, they report monthly to the State TBL supervisor who undertakes regular supervision visits to all the facilities. In accordance with WHO/NTBLCP guidelines, all pulmonary TB suspects submit three sputum specimens for microscopy and may or may not have an X-ray examination as part of their evaluation.

Within the public sector, TB services (consultations, diagnostic tests, drugs and hospitalisation) are provided free-of-charge. At the private (mission) hospitals, TB diagnostic tests and treatment services are also provided free-of-charge. However, all patients irrespective of their health problem visiting the facility pay a nominal consultation fee. Following diagnosis, very ill patients are admitted during the first month of treatment, whereas stable patients are treated as outpatients. TB patients admitted at the mission (private) hospitals pay a flat fee of $50–$67 for hospitalisation and food. Adherence is directly monitored by the health workers for admitted patients and during weekly drug collection visits for outpatients. In accordance with national guidelines, adherence for patients treated on an outpatient basis is observed by a family member (DOTS supporter) and/or a volunteer community health extension worker (CHEW) living in the patient's rural community. The CHEW keeps a record of the patients visited and treatment observations, and their activities are monitored by their district TB control supervisors.

Three rural hospitals; one secondary care public and two (not-for-profit) private from the three different zones in the State were selected as the study sites. The study sites were selected based on geographical spread, logistics and high TB notification rates. Together, they accounted for 70% of total TB notification in the state in 2009 [21], and, they each represent the main reference hospital for TB services in the three geopolitical zones in the state.

### Study design

In a cross-sectional cost-of-illness study, all new adult smear-positive or smear negative pulmonary TB patients who were registered in the three rural hospitals between January and August 2011 were eligible.

### Participants

Eligibility criteria: new pulmonary TB patients aged ≥15 years old, with at least one month but less than three months of treatment completed, and normally resident in Ebonyi State. Patients within this treatment period were included because they could easily recall their pathway to care.

### Sampling strategy & sample size

Consecutive consenting eligible patients were interviewed at each study site until the required sample size was reached. With a sample of 400 patients, we were able to detect an estimated 50% prevalence of TB patients with catastrophic costs at 95% confidence level and an absolute sampling error of 0.05.

### Data collection

Two locally recruited research assistants who are community health workers with bachelor's degrees and had participated in a standardised training session conducted the interview. A standardised questionnaire from the Poverty sub-working group of the StopTB Partnership titled: “*Tool to Estimate (TB) Patient's Costs*” was used [22]. Interviews were conducted in private rooms and the assistants translated the questions into the local language (primarily Igbo) according to patient preferences. Weekly supervision of the data collectors was conducted by district Tuberculosis and Leprosy Control supervisors and by the main researcher (UKN).

### Measurements and definitions

The primary outcome variable was the mean (median) patient and household costs incurred. Possible covariates included: age, gender, type of TB, household income group, household size, residence, category of health facility, and HIV status.

Four types of costs were summarised: direct including coping costs, indirect, guardian and household costs (Table 1). Also, costs were summarised for two periods: pre-diagnostic and post diagnostic (Table 1). Income lost pre-diagnosis was estimated from the difference in self-reported monthly patients' income before the illness and in the pre-diagnosis period. For time loss, we assumed that an average unskilled worker in Nigeria would work 24 days a month, 8 hours per day (224 hours a month), we used the monthly incomes reported by each patient to derive an hourly wage rate to estimate each patient's indirect pre-diagnosis costs from time loss. While for indirect costs post-diagnosis, we used minimum wage rate in Ebonyi State to derive wage rate for lost income and time – as most are poor patients who stopped working or suffered job losses during TB treatment, the least they could have earned during this period would have been the minimum wage in the State. However, for the few patients who retained their full pre-diagnosis monthly income, we used this to estimate their indirect costs post-diagnosis. All costs are reported in 2011 US$ (1 US$ = N150).

**Table 1:** Definitions used in the study.

Variable	Definition
Direct cost	Out-of-pocket payment for TB services and those incurred in the pathway to care to access the service. These include user fees, consultation fees, laboratory test fees, travel costs, food costs and other costs.
Indirect cost	Indirect costs are patients' lost income due to illness/time to receive care – including travel time, waiting time and time for consultation with a health worker.
Coping cost	Costs incurred by patients who attempted to cope with the costs of TB care by: borrowing money or selling their assets. It is estimated as sum of interest paid for loans and/or difference in the market value of assets sold.
Guardian cost	The costs incurred by family members looking after the patient during care. For each guardian, we summarised both direct and indirect costs.
Household cost	Household costs for each patient is the sum of patient direct, coping, indirect, and guardian costs.
Pre-diagnosis/diagnosis period	This was defined as the period between the onset of symptoms and the diagnosis of TB.
Post-diagnosis period	This was defined as the period from start to completion of intensive phase of treatment, approximately two months.
Poor patients	Patients who earned less than or equal to the median monthly income reported in the study $66.7 were classified as poor patients; while those who earned more than this median were classified as less-poor.
Poor households	Household which earned less than or equal to the median monthly income reported in the study ($133.3) were classified as poor households; while those who earned more than this median were classified as less-poor.

TB =  tuberculosis.

Routine data on sputum smear microscopy result, date of treatment initiation, HIV status, smear and type of TB obtained from the patients were verified from the TB treatment register and individual patient treatment cards.

### Data analysis

The data were double-entered, cleaned, and analysed using Epi Info 3.4.1 (CDC, Atlanta, GA USA). For each patient, cost summaries were also generated using Microsoft Excel (Microsoft, Redmond, WA, USA) and compared to ensure accuracy. Continuous variables were summarized as means (± standard deviation [SD]) or median (± interquartile range [IQR]) while categorical variables were summarized as proportions. Group comparisons were made using the χ^2^ test for proportions, t – test for means, Mann-Whitney test for medians in two groups.

Multivariable logistic regression analyses were used to determine factors associated with household poverty before TB illness, and after TB diagnosis and treatment. First, bivariate analyses were done with Chi-square tests to examine the effect of each predictor variable on household poverty. Then, a stratified analysis was performed to determine the occurrence of interaction and confounding between the predictor variables. Interactions were determined by Chi-square for differing odds ratios (ORs) by stratum, while confounding was assessed by comparing the Mantel-Haenszel summary OR to the crude OR. Multivariable models were then constructed; including all variables of clinical importance and all with bivariate *P*<0.2. A backward elimination approach was used to find the best model. P-values less than 0.05 were significant.

Sensitivity analyses were conducted using standard methods [23], to explore the degree of uncertainty associated with indirect cost estimates. We used a variation of 25% decrease/increase from the base-case scenario to explore the degree of uncertainty around length of pre-diagnostic return trips, number of drug collection visits, foregone income during treatment and foregone income post-diagnosis.

### Ethics Statement

The study was approved by the research and ethics committee of Ebonyi State University Teaching Hospital Abakaliki, Nigeria. Also, permission was obtained from the study sites. A signed informed consent was obtained from all patients before interview.

## Results

### Socio-demographic characteristics and care-seeking behaviour

The study surveyed 480 eligible patients. Full records were completed for 452 patients. The sample was 45% (203) female and 55% (249) male; 44% (198) of the patients were the primary household income earners, and 38% (170) had no formal education (Table 2). At the onset of symptoms, only 11% (48) of the patients initially visited a public facility; while 89% (404) reported that they first consulted either of a drug shop, mission hospital, traditional healer, or private hospital. For the latter group, factors which determined the patients' choice of facility for initial consultation were; waiting times 26% (118), costs 20% (90) and distance to the facility 23% (104). The median pre-diagnosis delay at the study facilities was 8 weeks (IQR 8, 12 weeks).

**Table 2:** Demographic, socio-economic and clinical characteristics of patients studied.

Variable	Total n (%)
**Age (years)**	
≤35	314 (69.5)
>35	138 (30.5)
**Household size (persons)**	
≤4	246 (54.4)
>4	36 (45.6)
**Education**	
None	170 (37.6)
Primary	180 (39.8)
Secondary	84 (18.6)
Graduate	18 (4.0)
**HIV Status**	
Positive	130 (29)
Negative	322 (71)
**Category of facility**	
Public	120 (26.5)
Private	332 (73.5)
**Residence**	
Urban	96 (21.2)
Rural	356 (78.8)
**Household income group**	
Poor	246 (54.4)
Less – poor	206 (45.6)
**Occupation**	
Subsistence farming	212 (46.9)
Small business	114 (25.2)
Casual labour	48 (10.6)
Housework	24 (5.3)
Civil servant	12 (2.7)
Other (student, transport e.t.c)	42 (9.3)
**Smear status**	
Smear positive	362 (80.1)
Smear negative	90 (19.9)
**First visited a public facility**	
Yes	48 (10.6)
No	404 (89.4)
**Median (IQR) pre-diagnosis delay in weeks**	8 (8, 12)

IQR =  inter-quartile range.

### Pre-diagnosis/diagnosis direct costs

Median direct (out-of-pocket) expenditures incurred pre-diagnosis was $49 ($39, $85) per patient (Table 3). This includes expenditures incurred due to consultations from public, private or informal providers. Few of the patients (n = 30) were admitted in the hospital before TB was diagnosed. Hospitalised patients incurred a substantial higher direct costs: hospitalised median ($103) versus not-hospitalised median ($45) (*P*<0.001). Before getting a TB diagnosis, patients completed a median of three health facility visits lasting a median of 10 hours.

**Table 3:** Total patients' and household costs of TB care and summary.

Timing and type of cost	US Dollar (IQR)	% of median annual HH*/patient income(Pre TB)
**PATIENT COSTS**		
**Median Direct Cost**		
Pre-diagnosis costs	48.7 (39.3, 85.3)	
Treatment costs (cost/visit x 8 visits)	36 (24, 42)	
Coping costs	26.7 (0, 50)	
Sub-Total	111.4	14
**Median Indirect Cost**		
Foregone income before diagnosis (months of work lost x difference in reported monthly income) +	333.3 (133.3, 800)	
Median value of return trips pre-diagnosis (hours)	2 (1.7, 3.3)	
Foregone income during treatment: drug collection time x minimum wage. (drug collection time = hours/visit x 8 visits) +	0.8 (0.3, 2.8)	
Months of work lost x minimum wage	80 (27.7, 93.3)	
Sub-Total	416.1	52
**Median Total Patient Costs**		
Direct + Indirect	527.5	66
**GUARDIAN COSTS**		
Median direct guardian cost	0 (0, 3.3)	
Median indirect guardian cost	48.5 (0, 96)	
Median total guardian cost	64 (0, 111.7)	
**HOUSEHOLD COSTS**		
Patient costs + guardian cost	591.5	37*

Pre-TB: median annual individual income  = $800; median annual household income $1, 600; AfterTB: median annual household income  = $1040; HH =  household; IQR =  Interquartile range; [All costs in 2011 US$].

Costs due to non-TB drugs and diagnostic tests accounted for a high proportion of direct expenditures in the pre-diagnosis period (36% and 28% respectively). The remaining proportion of the expenditures were for transportation (21%), food (7%) and user fees (8%) (Table 4). Patient pre-diagnostic costs did not differ significantly across smear status or gender. However, older age (*P* = 0.003), poor patients (*P* = 0.02), private hospital care (*P*<0.001), HIV-sero-positivity (*P*<0.001) and urban residence (*P*<0.001) were significantly associated with higher patient pre-diagnostic costs.

**Table 4:** Direct and indirect costs incurred by patients during the pre- and post-diagnostic periods.

Timing and type of cost	Patients reporting expenditure n (%)	Mean cost for all patients: ($)
**PREDIAGNOSIS**		
**Direct**		
Card/user fees	416 (92)	$6
Chest X-ray	300 (66.4)	$9
Laboratory test	258 (57.1)	$11
Non-TB drugs	416 (92)	$25
Transportation	446 (99)	$15
Accommodation	0	0
Special food	398 (88)	$4
Sub-total (direct)	452 (100)	$70
**Indirect**		
Value of time for return trips and clinic	452 (100)	$2
Foregone income before diagnosis	410 (91)	$511
Sub-total (indirect)	452 (100)	$513
**Total (mean) Pre-diagnosis**	452 (100)	$583
**POST-DIAGNOSIS**		
**Direct**		
Administrative	252 (44)	$1
Hospitalisation	30 (7)	$6
Food	252 (44)	$5
Transport (for drug collection and follow-up)	452 (100)	$28
Subtotal (direct)	452 (100)	$40
**Indirect**		
Value of time for drug collection visits	452 (100)	$0.8
Foregone income during treatment	452 (100)	$80
Subtotal (indirect)	452 (100)	$81
**Total (mean) post-diagnostic**	452 (100)	$121
**Total (mean) pre- and post- diagnostic cost**	452 (100)	$704

[All costs in 2011 US$].

### Post-diagnosis (treatment) direct costs

The patients surveyed had received anti-TB drugs for a median of 2 months of an 8-month regimen. Except for patients who were hospitalised during the first month of treatment, additional expenditures due to DOT visits were not incurred as all the surveyed patients received community-based DOTS at home. Median direct treatment cost was $36 ($24, $42) for 8 visits lasting a median of 140 (IQR 90, 200) minutes each (Table 3). Majority of the direct costs incurred during treatment were for transportation during drug collection/follow-up visits (70%) and food (13%); hospitalisation and user fees together accounted for (17%), (Table 4). Post-diagnosis costs did not differ significantly by HIV status, smear status or income level while, older patients (*P*<0.001), male patients (*P* = 0.002), private hospital care (*P*<0.001), or urban residence (*P*<0.001) had significantly higher costs.

### Coping costs

About 88% (398) of the patients adopted a coping strategy to handle the costs of TB; they either borrowed money only 212 (47%), sold assets only 42 (9%) or both 144 (32%). The median total amount for coping strategies was $26.7 ($0, $50) (Table 3). Poor patients were particularly likely to have borrowed money (*P*<0.02; χ^2^ = 6) or sold their assets (*P*<0.02; χ^2^ = 6) to cope with the costs of care. For patients who sold their assets, the amount earned was significantly below the market value estimated by the patient (*P*<0.001).

### Indirect costs

Indirect costs incurred during the pre-diagnostic period up to the end of the intensive phase of treatment ($416) constituted 79% of total patient costs ($528) or 52% of median annual patient income ($800). Most of the indirect costs were from lost income (Table 3). For the pre-diagnosis period, this was calculated using each patients' monthly income before the illness (median $66.7), multiplied by the duration of time the patient was out of work because of their illness (median 5 months, n = 410) i.e. median income loss equals $333.3; and added to the value of time loss (median 10 hours). For the post-diagnosis period, the monthly patient income – median $40 (i.e. the minimum wage in Ebonyi State) was multiplied by the duration of time on TB treatment (two months), and added to the value of the time loss over 8 drug collection visits (median 2.3 hours).

### HIV and TB

All the facilities surveyed had TB/HIV collaborative activities. About 29% (132) of the patients were HIV–infected. Compared with HIV-negative patients, HIV-positive patients incurred 43% higher direct costs during the pre-diagnosis period ($53 vs. $76; *P*<0.001). Also, post-diagnosis, HIV-positive patients incurred 11% higher direct treatment costs associated with collection of anti-retroviral drugs, however this was not statistically significant ($39 vs. $35; *P* = 0.6).

### Total patient and household costs

The sum of median direct and indirect patient costs was $528 (Table 3). This corresponds to 66% of median annual individual incomes ($800) before TB illness. The median household cost (patient and guardian) was $592 this was equivalent to 37% of median annual household income ($1600) before TB illness. After TB care, total household costs for TB care rose to 57% of median annual household income ($1040) (Table 3).

### Changes in household poverty level before and after TB

**Figure 1:**
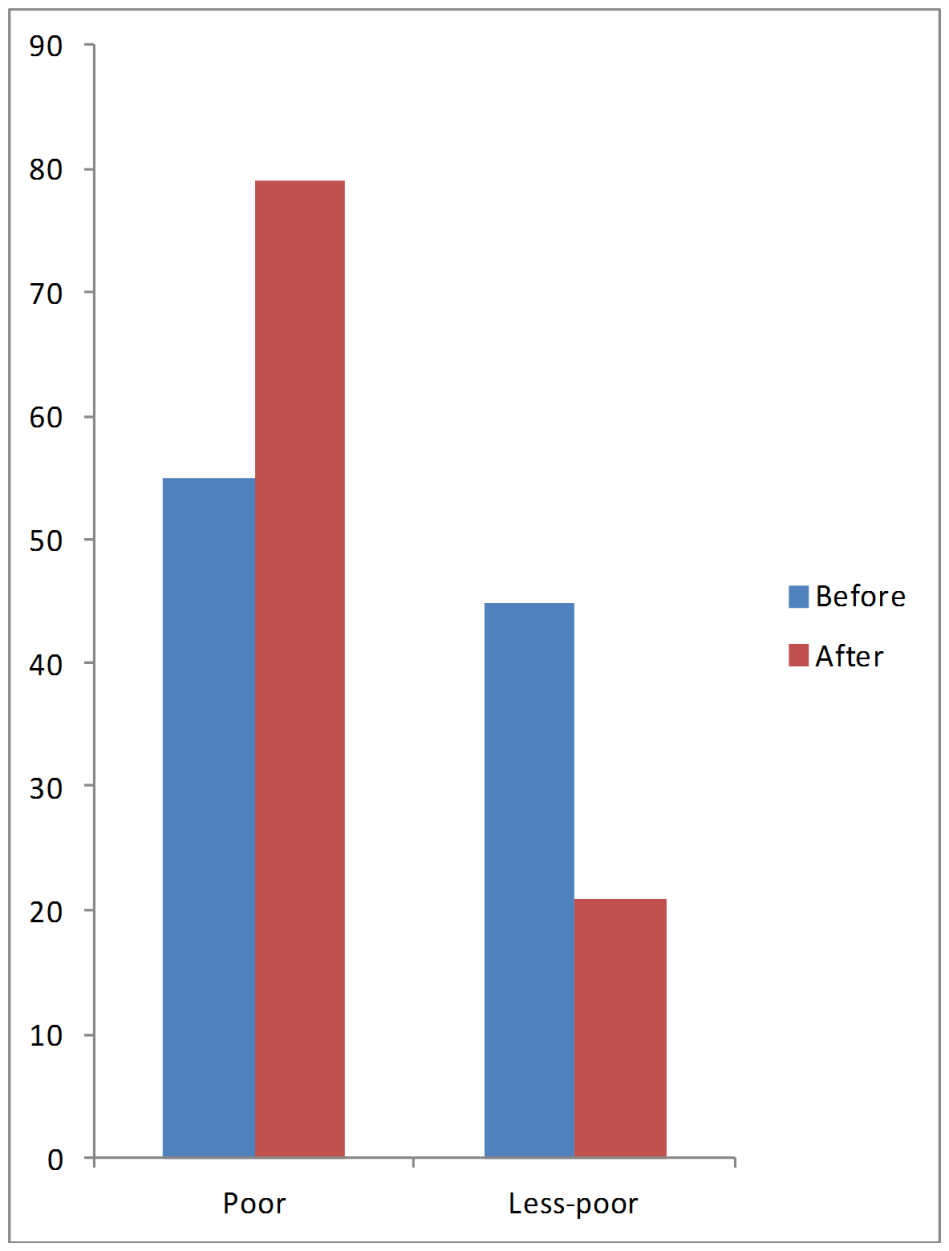
Household income changes before and after TB.

To inform the debate regarding increase in household impoverishment due to TB, we evaluated the effects of changes in household income on household poverty levels and its correlates. Following an episode of TB (before TB illness and after care comparison), there was a reduction in household income which increased the proportion of households classified as poor from 54% to 79% (Fig. 1). Across age-groups, residence and gender categories (Table 5), the proportion of households classified as poor following an episode of TB increased between 20% and 37% (P<0.001). Similarly, across type of facility visited, HIV status and smear status irrespective of category, the proportion of households classified as poor significantly increased following an episode of TB (Table 5). Furthermore, across patients' educational status, household where the patient with TB was a graduate were the most affected; in this group, households being classified as poor increased from 33% to 100% (P<0.001; Table 5).

**Table 5:** Changes in Household poverty level before TB illness and after TB care relative to patient demographics and clinical characteristics.

Variable	Total (N)	Before TB		After TB		P-value
		Poor n (%)	Less-poor n (%)	Poor n (%)	Less-poor n (%)	
Total	452	246 (54)	206 (46)	356 (79)	96 (21)	<0.001
**Age (years)**						
≤35	314	186 (59)	128 (41)	266 (85)	48 (15)	<0.001
>35	138	60 (43)	78 (57)	90 (65)	48 (35)	<0.001
**Gender**						
Male	248	132 (53)	116 (47)	206 (83)	42 (19)	<0.001
Female	204	114 (56)	90 (44)	150 (74)	54 (26)	<0.001
**Residence**						
Urban	96	42 (44)	54 (56)	78 (81)	18 (19)	<0.001
Rural	356	204 (57)	152 (43)	278 (78)	78 (22)	<0.001
**Education**						
None	170	108 (63)	62(37)	134 (79)	36 (21)	0.002
Primary	180	90 (50)	90 (50)	150 (83)	30 (17)	<0.001
Secondary	84	42 (50)	42 (50)	54 (64)	30 (36)	0.06
Graduate	18	6 (33)	12 (67)	18 (100)	0 (0)	<0.001
**HIV**						
Positive	132	96 (73)	36 (27)	114 (86)	18 (14)	0.006
Negative	320	150 (47)	170 (53)	242 (76)	78 (24)	<0.001
**Smear status**						
Positive	362	198 (55)	164 (45)	290 (80)	72 (20)	<0.001
Negative	90	48 (53)	42 (47)	66 (73)	24 (27)	0.005
**Category of facility**						
Public	120	42 (35)	78 (65)	90 (75)	30 (25)	<0.001
Private	332	204 (61)	128 (39)	266 (80)	66 (20)	<0.001

We further evaluated factors affecting household impoverishment before TB illness and after TB care (Table 6). Before TB illness, independent predictors of household poverty were; rural residence (adjusted odds ratio [aOR]; 95% confidence interval [CI], 2.8; 1.6–5.0), HIV-positive status (aOR 4.8; CI 2.7–8.3), and care-seeking at a private facility (aOR 5.1; CI 2.9–8.8). After TB care, independent determinants of household poverty were; younger age (≤35 years, aOR 2.4; CI 1.4–4.0), male gender (aOR 2.1; CI 1.3–3.5), and HIV-positive status (aOR 2.5; CI 1.3–4.7).

**Table 6:** Logistic regression analysis of factors affecting household impoverishment before TB illness and after TB care in Nigeria 2011.

	Before TB illness			After TB care		
Variables	Crude OR (95% CI)	Adjusted OR (95% CI)	Adjusted P-value	Crude OR (95% CI)	Adjusted OR (95% CI)	Adjusted P-value
**Age (years)**						
≤35	1.9 (1.3–2.6)	1.6 (1.0–2.5)	0.06	3 (1.9–4.7)	2.4 (1.4–4)	<0.001
>35	1			1	1	
**Gender**						
Male	1.1 (0.8–1.6)	1.2 (0.8–1.9)	0.4	1.8 (1.2–2.8)	2.1 (1.3–3.5)	0.005
Female	1	1		1	1	
**Residence**						
Rural	1.7 (1.1–2.7)	2.8 (1.6–5.0)	<0.001	1.2 (0.7–2.2)	1.1 (0.6–2.3)	0.7
Urban	1	1		1	1	
**Education**						
No	1.8 (1.2–2.7)	1.1 (0.7–1.9)	0.6	1.0 (0.6–1.6)	1.1 (0.6–2.0)	0.7
Yes	1	1		1	1	
**HIV**						
Positive	3.0 (1.9–4.7)	4.8 (2.7–8.3)	<0.001	2.1 (1.2–3.6)	2.5 (1.3–4.7)	0.005
Negative	1	1		1	1	
**Smear status**						
Positive	1.1 (0.7–1.7)	1.5 (0.9–2.5)	0.2	1.5 (0.9–2.5)	1.2 (0.6–2.2)	0.62
Negative	1	1		1	1	
**Category of facility**						
Private	3.1 (1.9–4.6)	5.1 (2.9–8.8)	<0.001	1.3 (0.8–2.2)	1.4 (0.8–2.5)	0.24
Public	1	1		1	1	

TB =  tuberculosis; OR =  odds ratio; 95% CI =  95% Confidence Interval.

### Productivity and social impact of TB

The survey sought to know the number of hours worked per week by each patient prior to and after the onset of TB symptoms. About 97% (440) reported that they worked fewer hours due to TB; (median hours worked per week decreased from 50 to 10). Of those who reported a reduction in the number of hours worked, a family member took over the job of 96% (422) of them; in 54% (228) of cases this was a child (68% female). Also, 83% (374) of respondents stated that their social life was affected due to TB, resulting in job losses 55% (258), dropping from school 11% (48), disruption of sexual life 11% (48), and separation/divorce from spouse 8% (36). None of the patients reported having any form of health insurance.

### Sensitivity analysis

A one-way sensitivity analysis (Table 7), showed that the results were not sensitive to possible imprecision in the estimated indirect costs for length of pre-diagnostic return trips, rate of drug collection visits, and foregone average income during treatment. However, indirect costs estimates were sensitive to changes in foregone income pre-diagnosis.

**Table 7:** Sensitivity analyses for varying differences of patients' length of pre-diagnostic return trips, length of foregone income pre-diagnosis, rate of drug collection visits, and average post-diagnostic income.

	Costs	Total (mean) patient costs
**Length of pre-diagnostic return trips**		
−25%	$1.8	$703.8
Base case (10 hours)	$2	$704
+25%	$2.5	$704.5
**Length and value of foregone income pre-diagnosis**		
Min (3 months)	$307	$300
Base case (5 months)	$511	$704
Max (7 months)	$715	$908
**Rate of drug collection visit**		
−25%	$0.6	$703.8
Base case (8)	$0.8	$704
+25%	$1	$704.2
**Average income foregone (post-diagnosis)**		
−25%	$60	$684
Base case	$80	$704
+25%	$100	$724

[All costs in 2011 US$].

## Discussion

In this study we have shown that in a setting of high burden of TB/HIV and poverty where TB services are free of charge, TB patients face considerable payments and financial losses before and during treatment. Most of the costs are due to lost income and time (52% of annual patient income pre-diagnosis). Thus, loss of work impacts economically on patients with TB resulting in impoverishment and worsening of their situations [14]. Also, we found that direct costs are still substantial (14% of annual patient income) with non-TB drugs, diagnostic tests, coping costs and transport as the biggest cost items [14], [16]. A few other studies from Africa have used similar instrument to assess TB patient costs [13]–[15]. The median direct patient costs observed in this study ($111) was far higher than the average direct costs of $7, $28 and $56 found in Zambia, Ghana and Kenya respectively [13]–[15]. Average patient costs observed as a proportion of their annual income are comparable to other studies which estimated the economic impact of TB in African countries [6], [9], [12]–[14]. Not surprisingly, a substantial proportion of TB patients adopted coping strategies that are likely to further impoverish their household to cope with the illness. Our finding was higher than previously observed in Tajikistan where 65.7% of patients relied on similar detrimental coping strategies to handle the costs of TB [24].

Also, we have shown that poor households with a TB patient face high economic burden of care [9]. Our results showed that irrespective of the household status, location, patient demographic or clinical characteristics, once TB occurs in a household, it drives the household deeper in to poverty [25], [26]. Our findings confirm a ‘medical poverty trap’ situation where expenses increased while incomes fell [14], [27]. Households with patients in the younger age group were the most associated with increased household impoverishment after TB care. A possible explanation is that this age group are the most productive economically [5]. Thus, any illness affecting households with younger patients are likely to suffer severe financial loses.

Also, TB/HIV co-infection was a significant predictor of increased out-of-pocket expenditures for TB care. This may be because of increased number of care-seeking visits before a proper diagnosis. A previous study suggests that collaborative TB/HIV services are cheaper for patients [7]; however, we found that although patient direct treatment costs did not differ according to HIV status, pre-diagnostic/diagnostic costs were significantly higher for HIV-infected patients. Our study suggests that collaborative TB/HIV services can be cheaper through decentralisation of these services to rural communities and by reducing patient pre-diagnosis delay.

Only one-tenth of the patients initially visited a public facility at the onset of TB symptoms. This led to long delays in treatment-seeking pathways before a proper diagnosis was made. Inefficiencies in public health facilities and lack of pro-poor TB control services at rural private facilities were two main constraints responsible for prolonged delay and increased cost of TB diagnosis [10], [13], [24], [28]. The rural poor are left with the private facilities who often charge user fees and other fees. This constrained choices of care for the poor results in TB being a significant predictor of increased household impoverishment among the poor [9]. Thus, interventions that ensure TB diagnosis closer to the homes of the patients, for example, through active community-based case finding could lower the household impact of TB before diagnosis. Also the existence of user fees and other fees indicates that although in theory TB services are provided free-of-charge, in practice it is impossible for an individual to receive these ‘free’ services without first paying to gain access to care [13].

TB case detection under the current DOTS strategy in Nigeria occurs through passive case finding [2]. Increasing patients and community education, removing user/accommodation fees, increasing health insurance coverage, and encouraging individuals to present early to health centres are strategies from our study that may improve TB case detection [13], [15]. There is also need to further strengthen the capacity of rural public facilities to offer TB services through improved health worker motivation and retention. In the absence of strong public health systems, the informal private sector must be strengthened to offer TB services in rural poor settings [29]. For example, linkages should be created with private drug shops/pharmacies to refer individuals with cough for more than 2 weeks to nearby facilities registered with the NTBLCP. Also, providing safe and quality-assured sputum collection points at strategic locations in the community for example, near the drug shops may ensure that individuals receive early diagnosis thereby lowering costs. More patient-support enablers, nutrition, and transport vouchers are other interventions that could lower patient and household costs [15].

Although gender was associated with higher costs in some studies [9], [13], consistent with a previous study, we found no difference in direct costs between male and female patients [24]. However, male gender was a significant predictor of increased household impoverishment after TB care. The most likely explanation for this finding is that men are the main primary income earners in the study setting. Thus, with their income lost due to TB illness and the direct costs for TB care-seeking, their household's may face catastrophic expenditures for their care. This also makes them prone to higher rates of default from treatment once they start improving following treatment as they try to return to work early [30]. A targeted social and economic intervention to households with a male TB patient may lower their risk of impoverishment.

Our study was limited by potential biases. First, we used a cross-sectional descriptive design and hence it is not appropriate to conclude that a statistical relationship constitutes a causal link. Second, as in other cost-of-illness studies, we relied on self-reported costs. Thus, recall and reporting bias cannot be excluded. We limited recall bias by conducting the interview within two months of starting treatment, which reduced recall time. Interviewers were trained to recognize unusually high costs for specific items and to clarify by comparing the reported costs to local prices for comparable items. In low-income countries, incomes are generally more difficult to estimate due to greater reliance on the informal economy, self-employment and seasonal/agricultural activity [14], [31]. Therefore, it is difficult to ensure that patients are reporting incomes. Comparing the annual per capita income of Nigeria in 2011 ($1200) [32], with our finding of median annual individual income of $800, it is likely that self-reported income was under-estimated in this study. But, given that the study was conducted among mainly the rural poor, the median income estimates are expected to be lower. In addition, asking individuals to assign monetary values to unpaid housework were difficult concepts to understand [14]. Another limitation is that we only analysed two coping strategies, while many more exist [24]. We focused on those financial coping strategies that may lead to future impoverishment.

In conclusion, patients and their households face major expenditures due to TB care and 88% of patients employ coping strategies that potentially impair future income. Also, costs of TB care accounted for 66% and 37% of patients and households income respectively. TB patients visit several informal providers and experience long delays before diagnosis in this setting. Improved community and patients' education about TB, strengthening of rural public and private health systems, incorporating informal health providers, removal of user fees and other fees, and further decentralization of TB/HIV collaborative activities to rural settings are simple interventions at the level of case management that could reduce diagnostic delays and lower expenditure for patients and their households. In view of the high costs, such mitigation strategies are urgently needed. Also, other interventions like financial protection specific for poor households and households with a male patient or HIV-positive individuals are needed. It is important to further investigate the impact of these interventions on patient costs and treatment outcomes within both the local context; and different low-income and rural settings.

## Supporting Information

Table S1 **Questionnaire used for the survey.**
(DOC)
