# Supplementary material for: The High Cost of Free Tuberculosis Services: Patient and Household Costs Associated with Tuberculosis Care in Ebonyi State, Nigeria
Source: PLoS One. 2013 Aug 27;8(8):e73134. doi: 10.1371/journal.pone.0073134 (PMC3754914; doi:10.1371/journal.pone.0073134)
Supplement: Table S1 — Questionnaire used for the survey. (DOC) [file pone.0073134.s001.doc]

**Project: Questionnaire and Consent Form**

**INFORMED CONSENT: Introduction to patients and consent**

Part 1: Introduction to the patient:

My name is………I am a research assistant working for Dr Ukwaja Kingsley who is conducting an academic project through the institution he is studying in, (EBSUTH). The project concerns the costs that people face when they are seeking health care. Therefore, we would like to inquire how much people spend on healthcare and more specifically on tuberculosis before and during diagnosis as well as during treatment.

It is important for you to understand that your participation in this study is completely voluntary. We would be really grateful if you would agree to participate in this study, but do feel free to refuse. If you refuse, there will be no consequence for you and you will receive whatever care and treatment you need at the health facility as usual. If you decline to participate you will not lose any benefit that you are entitled to such as receiving care and support that is provided at the clinic.

If you choose to participate in this study you need to know that you may withdraw from the study at any stage without giving any explanation for your withdrawal. Your answers will be kept confidential. At some point I will ask you about your personal income and the income of your household. We will NOT provide this information to any tax or welfare authorities, also not after the end of the study.

For more information, you can ask to see the Community Health Worker / Medical Officers in your health centres. In case of any inconveniences / ethical concerns about the study, you can contact the office of The Chairman, Ethics and Research Committee, Ebonyi State University Teaching Hospital, Abakaliki, Ebonyi State.

This survey will take approximately 20 minutes.

Part II: Certificate of Consent

**Do you have any questions? Do you want to participate?** (circle)  **Yes / No**

*If Yes:* Please give your consent as indicated below.

I have read the foregoing information, or it has been read to me. I have had the opportunity to ask questions about it and any questions that I have asked have been answered to my satisfaction. I consent voluntarily to participate as a participant in this research.

**Signature of Participant__________________ Date _____________________( Day/month/year)**

**If illiterate**

*A literate witness must sign (if possible, this person should be selected by the participant and should have no connection to the research team). Participants who are illiterate should include their thumb-print as well.*

**I have witnessed the accurate reading of the consent form to the potential participant, and the individual has had the opportunity to ask questions. I confirm that the individual has given consent freely.**

***Name / Signature of witness_____________________ AND Thumb print of participant***

**Date_*_____________________***

**Day/month/year**

**Researcher / Research Assistant**

I confirm that the participant was given an opportunity to ask questions about the study, and all the questions asked by the participant have been answered correctly and to the best of my ability. I confirm that the individual has not been coerced into giving consent, and the consent has been given freely and voluntarily.

**Name and signature of Researcher/person taking the consent_____________________**

**Date__________________________ (Day/month/year)**

**IF patient refuses consent by…..ask.** Is there a reason why consent for the study is not given?

1. *Language* not good enough 2. Time constraint 3. Not comfortable 4. Unspecified

**Patient registration Number :…………………. Questionnaire Number :………………**

| Date of Interview (dd/mm/yy) | Name of Province | Name of District | Place of interview (facility name) | Interviewer Name (Initials) |
| --- | --- | --- | --- | --- |
|  |  |  |  |  |
| Category of Facility | 1. General Hospital 2. Mission Hospital………………. | | | |

| *Patient Information (to be filled in by Interviewer with the help of patient card; fill in also if interview is refused for non-response analysis )* | | |
| --- | --- | --- |
| 1. Gender 1. male 2. female | | Age of patient: |
| 1. Type of TB (circle) | 1. pulmonary smear + 2. pulmonary smear - | |
| 1. Total duration of planned treatment (circle) | 1. (6 months) 2. (8 months) | |
| 1. Treatment Regimen (circle) | 1. Cat I (new Pulmonary) 2. Cat II (retreatment) | |
| 1. Currently in intensive or continuation phase? | 1. Intensive 2. Continuation | |
| 1. Interviewee | 1. Same as patient 2. DOT supporter / guardian 3. Other | |
| 1. HIV status   (only if indicated on card!) | 1. positive 2. negative | |

***Proceed according to inclusion/exclusion criteria previously defined!***

| 1. Date of Investigation (first sputum or xray dd/mm/yy) | Date of starting Treatment (dd/mm/yy) |
| --- | --- |

***Range of treatment duration required for this study is one month to three month*** *– proceed if in agreement with inclusion criteria.*

| *Previous Treatment* | |
| --- | --- |
| 1. a) Have you ever had TB treatment before?   *Cross-check with patient card; If No, go to 10.* | 1. Yes (mm/yy treatment ended) ……. ……. 2. No |
| b) If yes: Have you completed your previous TB treatment? | 1. Yes 2. No |
| c) If No: why not?  1. Lack of money for treatment costs 2. Drug side effects 3. Moved 4. distance to facility  5. Other (specify): | |

| ***Delay, Prediagnostic & Diagnostic Costs*** | |
| --- | --- |
| 1. **What symptoms did you experience that led you to seek treatment for your current illness? How long did you experience these symptoms before you went to seek treatment?**     1. Cough yes □ no □________ months 2. Night sweats yes □ no □ ________ months  3. Coughing up blood yes □ no □________ months 4. Weight loss yes □ no □ ________ months  5. Other (specify) yes □ no □ ________ months |  |

| 1. **Did you seek treatment or advice for these symptoms at any of the following?** *Check all that apply*   **Where did you go first?** *Circle first place of treatment*  1. District hospital yes □ no □ 2. Dispensary yes □ no □ 3. Health Centre yes □ no □  4. Mission hospital yes □ no □ 5. Pharmacy, drug & grocery store or chemist shop yes □ no □  6. Herbalist yes □ no □ 7. Private hospital/clinic yes □ no □ 8. Other (specify): □________  **b) Have you visited a traditional healer?** yes □ no □ |
| --- |

| *If other than public provider was chosen in 11):*   1. **Why did you not go to the public health facility, such as government clinic or hospital when you first realized you were sick?** *Circle most applicable.*   1. distance to facility 2. too expensive 3. time consuming to wait 4. lack of available facilities  5. mistrust of government health services provision 6. Belief system 7. No drugs available  8. other (specify) | |
| --- | --- |
| 1. **How far is the nearest government facility for**   **a) diagnosis and treatment**  **b) treatment only** | ____ hours walking ______ hours with transport other:__________  ____ hours walking ______ hours with transport other:__________ |

1. **About how much did you spend for each of these visits before you were diagnosed with TB, including the visit when you actually received your diagnosis?** *For all that don’t apply, mark N/A; Fill one line per visit*

|  | **Provider**  (copy from question xy providers where patient sought treatment or advice) | **Total Time spent**  **per visit**  (in hours, includes travel time) | **Administrative Costs** (consultative, registration) | **Test costs**  (for sputum  or other except xray) | **Xray costs (**includes sending xrays to radiologist, travel & fees) | **Drug costs**  (all kinds total) | **Travel Costs** (return total) | **Food costs** (total) | **Accomo-dation**  **Costs** (total) | **Sub-Total costs**  **per visit** | **Insurance Reimburse-ment**  *If yes: amount, if no n/a* |
| --- | --- | --- | --- | --- | --- | --- | --- | --- | --- | --- | --- |
| **Visit 1** |  |  |  |  |  |  |  |  |  |  |  |
| **Visit 2** |  |  |  |  |  |  |  |  |  |  |  |
| **Visit 3** |  |  |  |  |  |  |  |  |  |  |  |
| **Visit 4** |  |  |  |  |  |  |  |  |  |  |  |
| **Visit 5** |  |  |  |  |  |  |  |  |  |  |  |
| **Visit 6** |  |  |  |  |  |  |  |  |  |  |  |
| **Visit 7** |  |  |  |  |  |  |  |  |  |  |  |
| **Visit 8** |  |  |  |  |  |  |  |  |  |  |  |
| **Visit 9** |  |  |  |  |  |  |  |  |  |  |  |
| **TOTAL** | --------------------------------------- |  |  |  |  |  |  |  |  |  |  |

| **Total Direct Prediagnostic & Diagnostic costs (sum sub-totals) minus insurance =**  ***Name of currency*** |
| --- |

| ***Treatment Costs*** | | |
| --- | --- | --- |
| *Costs related to DOT* | | |
| 1. **Where do you currently take your TB drugs?**   *If the patient has visited two different DOT places, tick the current place and report costs only for that place.*  *If DOT at home, go to 19.*  1. Health facility / hospital 2. Home 3. Community 4. Workplace 5. Dispensary  **15 b) How many times per week do you go there place to take your drugs?**  □ 3 times □ 5 times □ 6 times □other | | |
| 1. **How long does it take you to get there** (one way) | ____ hours walking ______ hours with transport other:__________ | |
| 1. **How long does one of these visits take on average, including time on the road and waiting time** (total turnaround time)**?** | | Hours |
| 1. **From your home to the DOT place, how much does it cost if you take transport?** (both ways) | |  |
| 1. **How much do you spend on food on the road, while waiting, for lunch?** | |  |

| *Costs related to picking up the TB drugs – where drugs are currently picked up* | | |
| --- | --- | --- |
| 1. **How often do you travel to the health facility / hospital for picking up your TB drugs?** | | Times / month |
| 1. **How long does it take you to get there** (one way) | ____ hours walking _____ hours with transport ______ other | |
| 1. **How long does one of these visits take on average, including time on the road**   **and waiting time** (total turnaround time) ? | | hours |
| 1. **From your home to the facility, how much does it cost if you take transport?** (both ways) | |  |
| 1. **If you go to a facility to pick up your drugs, how much do you spend on food on that day?** (on the road, while waiting, lunch etc.) | |  |
| 1. **a) Do you have to pay administration fees when picking up your TB drugs?**   *If No, go to 26.*  **b)** If YES, **how much?** | | 1. Yes 2. No |
| 1. **a) Do you have any accommodation costs when picking up your TB drugs?**   *If No, go to 27.*  **b)** If YES**: how much?** | | 1. Yes 2. No |

| *Costs related to follow up tests* | |
| --- | --- |
| 1. **a) Did you ever have to go to the health facility in addition to your regular visits for follow up tests since the beginning of treatment?** *If No, go to 28.*   **b) If yes, how many times?**  **c) If yes, did you have to pay any additional costs any time during the entire period?**  **d) If so, what kind of costs and how much?** Fees_______ sputum test ________  Xray______ TB Drugs _______ Other Drugs________ Other_________ | 1. Yes 2. No  Times  1. Yes 2. No  Total: |
| **e) How long does one of these follow-up visits take on average, including time on the road, waiting time and tests** (total turnaround time) **?** | Hours |

| ***Guardian Costs*** | |
| --- | --- |
| 1. **a) Does any family/friend/DOT supporter accompany you on any visits or go in your place to collect your TB drugs** ? *If No, go to 29.* | 1. Yes 2. No |
| **b)** *If YES,* **on how many visits has your family/friend/DOT supporter accompanied you or gone in your place?** *Record pre-diagnosis/diagnosis visits and treatment visits separately*  Complete at data entry:  Pre-diagnosis/diagnosis costs per visit: Transport ______ Food ______ Accommodation ____  Costs during treatment per visit: Transport _____ Food ______ Accommodation __________ | _____ Diag. times  ______Treatment times  Total Diag:  Total Treatm: |
| **c) How much does your friend/family/DOT supporter earn per day?** | 1.  2. Doesn’t earn |
| **d)** **Why did someone accompany you?**  1. Distance 2. Security 3. Administrative barriers 4. Too ill to travel alone    5. Was required for treatment 6. Other (specify) | |

| ***Hospitalization*** | |
| --- | --- |
| 1. **Have you been hospitalized before or during your TB treatment?**   *If No, go to question 38.* | 1. Yes 2. No |
| 1. If YES: **how many days in total did you stay at the hospital?** | days |
| 1. **How much did you pay in the hospital during your entire stay?**   Hospital administration fees: Sheets/Linnen:  Food (not provided by hospital): Transport (return):  Drugs: Tests: Others: | Total: |
| 1. **Did any family/friend stay with you while in hospital?** *If No, go to question 38.* | 1. Yes 2. No |
| 1. If YES**: How many days did he/she stay with you (sleep there)?** 2. **Were there any extra costs for your relative/friend for staying at the hospital?**   Accommodation (hospital or other): Food:  Transport: Other: | Days  1. Yes 2. No  Total Costs: |
| 1. **How much does your friend/family normally earn per day?** | 1.  2. Doesn’t earn |
| 1. **a) Did any other family/friend visit you while in hospital?** *If No, go to 38.*   **b) If yes, how many people visited you?**  **c) how many times did they visit you?**  Accommodation per person: Food per person:  Transport per person: Other:   1. **How long were the visits including traveling time?** | 1. Yes 2. No  Persons  Times  Total number of visits:  Total Cost per person:  hours |

| ***Other Costs*** *Food Supplements* | | |
| --- | --- | --- |
| 1. **a) Do you buy any supplements for your diet because of the TB illness, for example vitamins, meat, energy drinks, soft drinks, fruits or medicines?** *If No, go to 39.* | | 1. Yes 2. No |
| **b)** If YES: **What kind of items?** (specify)  1. Fruits 2. Drinks 3. Vitamins/Herbs 4. Meat 5. Other (specify): | | |
| **c)** **How much did you spend on these items in the last month approximately?** |  | |

| ***Other Illnesses*** | |
| --- | --- |
| 1. **a) Do you have any chronic illness for which you are receiving treatment?**   *If No, go to 40.*  **b)** If yes: **which?** | 1. Yes 2. No |
| **c)** **Are there any additional costs for you because of this other illness besides the costs that you have already mentioned?** *If No, go to 40.* | 1. Yes 2. No |
| **d)** If YES: **How much are these additional costs on average per month?**  Tests: Drugs: Transport: Food:  Other: | Total: |
| 1. **How much did you spend on healthcare on average per month BEFORE the TB illness?** |  |
| 1. **How much do you spend on healthcare on average per month NOW?** |  |

| ***Insurance*** |  |
| --- | --- |
| 1. **a) Do you have any kind of private or government health/medical insurance scheme?**   *If No, go to 43.* | 1. Yes 2. No |
| **b)** If YES: **What type?**  1. reimbursement scheme 2. monthly medical allowance 3. donor  4. family/community fund 5. Western scheme (contract) 6. Other (specify) | |
| **c)** **Have you received reimbursement for any costs related to the TB illness?**  *Cross-check with question xy (table on prediagnostic & diagnostic costs) If No, go to 43.* | 1. Yes 2. No |
| **d) How much have you received as reimbursement?** |  |

| ***Coping Costs*** | |
| --- | --- |
| 1. **Did you borrow any money to cover costs due to the TB illness?**   *If No, go to question 45.* | 1. Yes 2. No |
| 1. **a)** If YES: **How much did you borrow?**   **b) From whom did you borrow?** *Circle most appropriate*  1. Family 2. Neighbors/friends 3. Private bank 4. Cooperative  5. Other (specify): |  |
| **c)** **What is the interest rate on the loan? (%)**  1. less than 10% 2. 10 to 20% 3. More than 30% 4. I don’t pay any interest  5. I am not expected to pay back the money | |
| 1. **a) Have you sold any of your property to finance the cost of the TB illness?**   *If No, go to 46.* | 1. Yes 2. No |
| **b)** If YES**: What did you sell?** *Circle most appropriate*  1. Land 2. Livestock 3. Transport/vehicle 4. Household item 5. Farm produce  6. Other (specify): | |
| **c) What is the estimated market value of the property you sold?** |  |
| **d) How much did you earn from the sale of your property?** |  |

| ***Socioeconomic Information***  *Individual Situation and Income* | |
| --- | --- |
| 1. Who is the primary income earner in the household? *Circle most appropriate*   1. Patient 2. Wife/mother 3. Husband/father 4. Extended family 5. Son/daughter  6. Other (specify) |  |
| **What is the highest level of education of** ...?   1. **The patient?**   1. Not attended/illiterate 2. primary 3. secondary 4.graduate/certificate 5. other   1. **Primary income earner?**   1. Not attended/illiterate 2. primary 3. secondary 4.graduate/certificate 5. other   1. **Head of household?**   1. Not attended/illiterate 2. primary 3. secondary 4.graduate/certificate 5. primary income earner = head of hh   1. Spouse of head of household? *If more than one spouse, choose highest level of education*   1. Not attended/illiterate 2. primary 3. secondary 4. graduate/certificate 5. other |  |

| 1. Are you currently formally employed?   *Name all options first* | 1. Yes, formal work *(go to 54)* 7. Combination (specify)  2. No, informal work *(go to 54)*  3. On sick leave *(go to 52)* 8. Other (specify)  4. Retired *(go to 52)*  5. School, university *(go to 58 )*  6. Housework *(go to 54 )* | | |
| --- | --- | --- | --- |
| 1. **Is the reason for Not Working related to the TB illness?** | | | 1. Yes 2. No |
| 1. If Yes**: When was the last time you were working? (mm/yy)** | | |  |
| 1. How are you usually paid?   1. cash 2. in kind 3. cash and in kind 4. not paid 5. bank transferred salary 6. other | | | |
| 1. What was your estimated personal take home earning per month BEFORE the TB illness? (includes welfare, disability, or other social support):   1. Under N10, 000 per month 2. N10, 000 to 20,000 per month 3 N20, 000 to 30, 000 per month 4. More than N40, 000 per month 5. Don’t earn | | | |
| 1. What is your estimated personal take home earning per month NOW? (includes welfare, disability, or other social support)   1. Under N10, 000 per month 2. N10, 000 to N20, 000 per 3. N20, 000 to N30, 000 per month 4. More than N40, 000 per 5. Don’t earn | | | |
| *If answer to 56 differs from 55:*   1. Is the change related to the TB illness? | | 1. Yes 2. No | |

| 1. **a) Have you ever stopped working/going to school/doing housework due to TB?** *If No, go to 59.* | | | | | | 1. Yes 2. No | |
| --- | --- | --- | --- | --- | --- | --- | --- |
| **b)** If YES**: for how long?** | 1. Less than 1 month 2. one month 3. 2-3 months 4. 4-5 months 5. more than 6 months | | | | | | |
| 1. **a) Does someone stay home specifically to take care of you?**   *If NO, go to 60*  **b**) If YES: **for how long?**  **c) Did they quit their income-earning job to stay home and care for you?** | | | | | 1. Yes 2. No  Weeks  1. Yes 2. No | | |
| 1. How regularly did you work before you became ill with TB? | | 1. Throughout the year 2. Seasonal/part of the year 3. Day labor 4. Other | | | | | |
| 1. Did you have to change jobs   when you became ill with TB? | | 1. Yes 2. No | | | | | |
| 1. What is your main occupation? *Tick all that applies, cross-check with question 51.*   1. Sales/Service 2. Agriculture 3. Household 4. Production/construction  5. Combination (specify) 6.Other (specify) | | | | | | | |
| 1. How many hours did you work on average per day BEFORE you became ill with TB? | | | | | | | Hours |
| 1. How many hours do you work on average NOW per day? | | | | | | | Hours |
| *If answer to 64 differs from answer to 63:*   1. Is the change related to the TB illness? | | | 1. Yes 2. No | | | | |
| *If answer to 64 differs from answer to 63:*   1. a) Is someone doing the work that you used to do?   **b)** 1. daughter 2. son 3. spouse 4. friend 5. nobody 6. other family | | | | | | | |
| 1. a) Do you have children of or below school age? *If No, go to 68.* | | | | | | 1. Yes 2. No | |
| b) Do all of your children of school age attend school regularly? *If YES, go to 67d)* | | | | | | 1. Yes 2. No | |
| c) If NO: Why not? *Circle most appropriate*  1. Needs to help around the house 2. No money for school fees 3. Also sick 4. Has to work to earn income  5. Other (specify): | | | | | | | |
| **d) Do any of your children of or below school age work to finance costs**  **due to the TB illness?** | | | | | | 1. Yes 2. No | |
| 1. **If you employed someone to do the housework for your household,**   **how much would you have to pay him/her per day?**   1. **While you are sick** 2. **While you are healthy** | | | | | |  | |
| 1. Are you financially independent? | | | | | | 1. Yes 2. No | |
| 1. a) Has the TB illness affected your social or private life in any way? *If No, go to 71.*   1. No 2. Divorce 3. Loss of Job 4. Dropped out of school 5. Separated from spouse/partner  6. disruption of sexual life 7. Sick child 8. Other (specify): | | | | | | | |
| b) If Yes: Has this resulted in a financial burden? | | | | 1. Yes 2. No | | | |
| 1. *What is your tribe / ethnic group / religion?* | | | | 1. 2. 3. 4. 5. | | | |

| *Household Income and Spending* | | | |
| --- | --- | --- | --- |
| 1. How much do you estimate was the average income of your household per month BEFORE the TB illness ? (for all persons in the house, including patient; includes welfare payments, government assistance or other social support)   1. income patient: 2. income rest of household 3. welfare payments  4. government assistance 5. Other: TOTAL: | | | |
| 1. How much do you estimate is the average income of your household per month NOW ?   1. income patient: 2. income rest of household 3. welfare payments  4. government assistance 5. Other: TOTAL: | | | |
| 1. How many people regularly sleep in your house? (including patient)   *If patient lives alone, go to question 77 and replace the word ‘household’ with ‘you’* | |  | |
| 1. How many of the household members are paid for working? (including patient) (*includes payment in kind or farm produce)* | |  | |
| 1. a) Besides yourself, does anyone else of your household receive treatment for TB? *If No, go to 77.* | | 1. Yes 2. No | |
| b) If Yes: How many? | |  | |
| 1. How much food did your household consume every month on average BEFORE the TB illness?   *Calculate value*  *If home production:*  If the food that you consumed per month before the TB illness was sold on the market: How much would it be worth? (plus how much you spent on average on food not produced at home?) | | |  |
| 1. How much food does your household consume NOW every month on average? *Calculate value*   *(for same number of people)*  *If home production:*  If the food that you consume per month now was sold on the market: How much would it be worth? (plus how much you spent on average on food not produced at home?) | | |  |
| 1. *If answer to 78 differs from 77:* Has the amount of food consumed per month changed due to the TB illness? | 1. Yes 2.No | | |

| ***Socioeconomic Indicators*** | | | |
| --- | --- | --- | --- |
| 1. What is your electricity supply? | | 1. Own connection 2. Shared connection 3. None | |
| 1. What is your source of drinking water?   1. Rainwater 2. lake/pond/ dam/river 3. public well 4. private well/bore hole 5. piped water 6. bottled water | | | |
| 1. What type of toilet facility is available?   1. no facility/bush/field 2. shared pit toilet/latrine 3. own pit toilet/latrine 4. flush toilet | | | |
| 1. How many rooms are there in your house?   1. 1 room 2. 2 rooms 3. 3 rooms 4. 4 or more rooms | | | |
| 1. Current place of residence? | 1.Urban 2. Urban Slum 3. Rural 4. Other (specify) | | |
| 1. Do you own the house or residence you live in? | | | 1. Yes 2. No |
| 1. Do you own….   *Include standard assets adapted to country Demographic and Health Survey (DHS)*   1. mobile phone 2. washing machine 3. motorcycle 4. bicycle 5. land (quantify-plots) 6. etc… | | | |
| 1. If the government could provide you with some service to ease the burden of TB on you and your household, what would you prefer to have? *State options, choose one*    1. Transport vouchers 2. food vouchers 3. More efficient service 4. Other (specify): | | | |

We would like to know the cost of the TB illness on the welfare of your household; that is, we would like to put a value on the TB illness which includes pain and suffering.

Therefore, we would like to know how much it would be worth to you if you could avoid becoming ill with TB in the first place. Note that we don’t ask what you actually can, but what you would be willing pay if you had an unlimited amount of money.

| 1. **How much would you be willing to pay for not becoming ill with TB in the first place?**   1. Under N20, 000 2. Between N20, 000 and N50, 000 3. Over N50, 000 4. Other (specify) |
| --- |

**Thank you for your cooperation! Is there anything you would like to ask or say?**

**Comments by Interviewer:**

**Date, Signature by Interviewer:**
